# Getting the Grip on Nonspecific Treatment Effects: Emesis in Patients Randomized to Acupuncture or Sham Compared to Patients Receiving Standard Care

**DOI:** 10.1371/journal.pone.0014766

**Published:** 2011-03-23

**Authors:** Anna Enblom, Mats Lekander, Mats Hammar, Anna Johnsson, Erik Onelöv, Martin Ingvar, Gunnar Steineck, Sussanne Börjeson

**Affiliations:** 1 Division of Nursing Science, Department of Medical and Health Sciences, Linköping University, Linköping, Sweden; 2 The Vårdal Institute, Lund, Sweden; 3 Department of Clinical Neuroscience, Osher Centre for Integrative Medicine, Karolinska Institute, Stockholm, Sweden; 4 Stress Research Institute, Stockholm University, Stockholm, Sweden; 5 Department of Clinical and Experimental Medicine, Obstetrics and Gynecology, Linköping University, Linköping, Sweden; 6 Division of Physiotherapy, Department of Oncology, University Hospital, Lund, Sweden; 7 Division of Clinical Cancer Epidemiology, Department of Oncology, Karolinska Institute, Stockholm, Sweden; 8 Division of Clinical Cancer Epidemiology, Department of Oncology, Sahlgrenska Academy, Gothenburg, Sweden; 9 Centre of Surgery and Oncology, Linköping University Hospital, Linköping, Sweden; Cedars-Sinai Medical Center and University of California Los Angeles, United States of America

## Abstract

**Background:**

It is not known whether or not delivering acupuncture triggers mechanisms cited as placebo and if acupuncture or sham reduces radiotherapy-induced emesis more than standard care.

**Methodology/Principal Findings:**

Cancer patients receiving radiotherapy over abdominal/pelvic regions were randomized to verum (penetrating) acupuncture (n = 109; 99 provided data) in the alleged antiemetic acupuncture point PC6 or sham acupuncture (n = 106; 101 provided data) performed with a telescopic non-penetrating needle at a sham point 2–3 times/week during the whole radiotherapy period. The acupuncture cohort was compared to a reference cohort receiving standard care (n = 62; 62 provided data). The occurrence of emesis in each group was compared after a mean dose of 27 Gray. Nausea and vomiting were experienced during the preceding week by 37 and 8% in the verum acupuncture group, 38 and 7% in the sham acupuncture group and 63 and 15% in the standard care group, respectively. The lower occurrence of nausea in the acupuncture cohort (verum and sham) compared to patients receiving standard care (37% versus 63%, relative risk (RR) 0.6, 95 % confidence interval (CI) 0.5–0.8) was also true after adjustment for potential confounding factors for nausea (RR 0.8, CI 0.6 to 0.9). Nausea intensity was lower in the acupuncture cohort (78% no nausea, 13% a little, 8% moderate, 1% much) compared to the standard care cohort (52% no nausea, 32% a little, 15% moderate, 2% much) (p = 0.002). The acupuncture cohort expected antiemetic effects from their treatment (95%). Patients who expected nausea had increased risk for nausea compared to patients who expected low risk for nausea (RR 1.6; Cl 1.2–2.4).

**Conclusions/Significance:**

Patients treated with verum or sham acupuncture experienced less nausea and vomiting compared to patients receiving standard care, possibly through a general care effect or due to the high level of patient expectancy.

**Trial Registration:**

ClinicalTrials.gov NCT00621660

## Introduction

Many cancer patients express interest in acupuncture for nausea [1]-[2] but it is not known if acupuncture is more effective for emesis (nausea and vomiting) than standard care during radiotherapy. Approximately 60% of patients irradiated over abdominal and/or pelvic fields experienced emesis during radiotherapy [1], [3]–[4]. Antiemetics are effective, especially serotonin-receptor antagonists combined with corticosteroids [5]. However, some patients at risk for nausea do not receive potent antiemetics, do not respond satisfactorily [1], [3], [5], or experience side-effects [5]. In a previous study we found that of 145 nauseous patients irradiated over a variety of regions, one third asked for more treatment against nausea while 40% rejected antiemetics [1].

Between two and 31% of patients undergoing cancer treatment use acupuncture for various kinds of symptoms [2]. In chemotherapy-induced nausea, acupuncture and acupressure reduced nausea more than antiemetics, but those studies did not include any sham treated control groups [6]–[11]. In a study of 80 chemotherapy patients, penetrating acupuncture did not reduce nausea more than telescopic non-penetrating sham needles [12]. In our study of radiotherapy-induced nausea, 70% of patients randomized to penetrating acupuncture and 62% of patients treated with telescopic sham needles experienced nausea during the radiotherapy period [13]. Apparently there was a lack of effects that could be related to the specific characteristics of verum (genuine) acupuncture; i.e. stimulation of skin penetrating needles in traditional acupuncture points resulting in a “deqi” sensation. However, as many as 95% of patients in *both* groups considered the treatment to be effective, and 89% were interested in receiving the treatments in the future [13]. In the light of the apparent conflict between lack of specific effects from verum acupuncture and large subjectively experienced positive effects it seems interesting to evaluate if acupuncture has antiemetic effects related to nonspecific mechanisms.

The aims of the study were to compare nausea and vomiting experienced by a cohort treated with verum or sham acupuncture with that experienced by a cohort receiving standard care during radiotherapy, and to evaluate if expectations of nausea and of acupuncture effects were related to the actual occurrence of nausea.

## Materials and Methods

The protocol for this trial and supporting CONSORT checklist are available as supporting information; see Protocol S1 and Checklist S1.

### Inclusion

**Figure 1:**
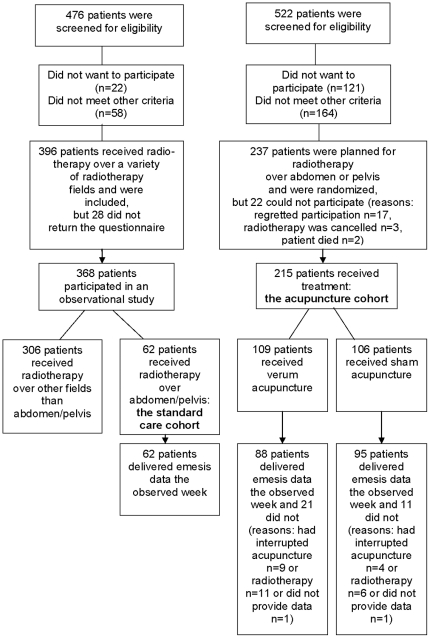
Selection of the patients in the standard care and the acupuncture cohort.

Two cohorts of patients treated for cancer in three Swedish oncology departments were included: one a standard care and the other an acupuncture cohort, see figure 1. The standard care cohort was created by a cross-sectional selection in four different days at two oncology departments in 1999 and 2003 [1] (n = 62). The acupuncture cohort was created from consecutively included patients in 2004 to 2006 at one of the two oncology departments referred to above and also in another oncology department [13]. Members of this cohort were randomized to verum acupuncture (n = 109) or sham acupuncture (n = 106). Inclusion criteria for both cohorts were that patients were at least 18 years of age, had radiotherapy over abdominal or pelvic fields and were able to take part in the study procedure. Exclusion criteria for the acupuncture cohort only were radiotherapy of less than 800 cm^3^ volume and 25 Gray dose, antiemetic treatment or persistent nausea within 24 hours prior to the start of radiotherapy and acupuncture treatment during the past year for any indication or ever for nausea.

All patients gave their informed written consent and the Regional Ethical Review Board in Linköping, Sweden, approved the study. The informed consent form used in the acupuncture cohort contained the information: “You will receive an ordinary acupuncture treatment with needles penetrating the skin or another treatment with needles placed just against the skin”. The study-evaluator and all health-care professionals, with the exception of the acupuncture-providing therapists, were blind to the acupuncture allocation. The standard care group knew, of course, that no acupuncture was given. They had been informed that the aim of the data collection was to evaluate the prevalence of nausea during radiotherapy.

### Treatment regimens

The acupuncture and the standard care cohort were, except for study participation, treated according to clinical routines, including the use of rescue antiemetics. The standard care cohort received no acupuncture therapy. One physiotherapist at each hospital (performing 1412 and 607 treatments) performed both verum and sham acupuncture and they had five deputy physiotherapists (performing 228, 75, 54, 32 and 6 treatments). Treatments started on the first day of radiotherapy, continued 30 minutes per session three times/week for two weeks, and then twice/week, until the end of radiotherapy according to a standardised treatment protocol. The patients were in a hotel, ward unit or at the radiotherapy department during the treatments, received either in a sitting or a supine position. The physiotherapists treated one to three patients simultaneously and maintained an everyday conversation, but avoided the subject of nausea.

*Verum* acupuncture was administered bilaterally to the traditional antiemetic point pericardium six (PC6) [14] between the tendons of palmaris longus and flexor carpii radialis at two body-inches proximal of the wrist crease. Sharp needles, diameter 0.30 × length 40 millimetres, were inserted into a depth of a half body-inch. One body-inch (or a “cun”: approximate 1.5 cm) is equivalent to the greatest width of the individual patient's thumb at the distal phalanx. The needles were manipulated three times/treatment by twirling and lifting until “deqi” occurred. “Deqi” is the specific sensation of verum acupuncture, involving heaviness, numbness, soreness and a minimal muscular contraction around the needle [15].

*Sham* acupuncture was administered bilaterally to a sham point located two body-inches proximal to PC6, outside traditional acupuncture points. “Park's sham devise” [16], 0.30×40 millimetres (extended length) was used. The credible [13] blunt telescopic needle glides upwards into its handle instead of penetrating the skin, and thus gives the illusion of penetration. Double-sticky marking tubes, used in both groups, held the sham needles in place. The therapists manipulated the sham needles three times/session until the needles touched the skin, but no “deqi” occurred. The duration of needle pressure to the skin was approximately ten seconds/session.

### Data collection

#### Background data

Clinical data, listed in table 1, were extracted from the patients' medical records. Other background variables, listed in table 2, were collected in a written questionnaire.

**Table 1:** Clinical characteristics of the patients in the verum acupuncture, sham acupuncture or standard care group.

Characteristics	Acupuncture cohort n = 215		Standard care cohort n = 62	Experiencing nausea n = 172/total n providing data = 267^1^	Univariable relative risk (95 % confidence interval)	Multivariable^2^ relative risk, (95 % confidence interval) adjusted for three groups
	Verum acupuncture n = 109	Sham acupuncture n = 106				
**Tumor diagnose**, n (%)	n = 109	n = 106	n = 62	n = 267		
Gynecological-	72 (66)	75 (71)	37 (60)	111/178 (62)	1.0 (Ref.)	1.0 (Ref.)
Colon-/rectal-	31 (28)	29 (27)	11 (18)	43/67 (64)	1.0 (0.8–1.3)	1.0 (0.8–1.3)
Testicular-	2 (2)	0 (0)	6 (10)	7/8 (88)	1.4 (1.1–1.8)	1.5 (1.0–2.2)
Pancreas, stomach or gallbladder-tumor	4 (4)	2 (2)	8 (13)	11/14 (79)	1.3 (0.9–1.7)	1.3 (0.9–1.9)
**Total radiotherapy dose** (Gray) mean ± SD	47.9 ±10.7	50.3 ± 10.3	41.8 ± 10.0	47.3 ± 10.5		
**Concomitant chemotherapy**, n (%)	n = 100	n = 99	n = 61	n = 260		
Yes	28 (28)	29 (29)	15 (25)	57/72 (79)	1.3 (1.1–1.6)	1.3 (1.1–1.6)
No	72 (72)	70 (71)	46 (75)	112/188 (60)	1.0 (Ref.)	1.0 (Ref.)
**Consumption of antiemetics at least once**, n (%)	n = 100	n = 101	n = 62	n = 263		
No	67 (67)	69 (68)	36 (58)	74/162 (46)	1.0 (Ref)	1.0 (Ref)
Any type	42 (42)	37 (37)	26 (42)	98/105 (93)	2.1 (1.7–2.4)	2.0 (1.7–2.4)
Serotonin-receptor antagonists	21 (21)	23 (23)	7 (11)	48/51 (94)	2.1 (1.7–2.5)	1.6 (1.2–2.0)
Dopamine-receptor antagonists	24 (24)	21 (21)	6 (10)	48/51 (94)	2.1 (1.7–2.5)	2.1 (1.7–2.6)
Corticosteroids	13 (13)	25 (25)	1 (2)	34/39 (87)	1.9 (1.6–2.3)	1.9 (1.5–2.3)
Antihistamines or neuroleptics	12 (12)	9 (9)	18 (29)	37/39 (95)	2.1 (1.7–2.5)	1.6 (1.3–2.0)
**Medication for any other illness/symptom**, n (%)	n = 99	n = 100	n = 62	n = 261		
Yes	80 (80)	88 (88)	40 (65)	140/208 (67)	1.2 (0.9–1.5)	1.2 (0.9–1.6)
No	19 (19)	12 (12)	22 (35)	30/53 (57)	1.0 (Ref.)	1.0 (Ref.)

Numbers (n) of patients answering the questions are presented, ^1^267 of 277 patients provided data regarding nausea. Experiencing nausea was defined as any day within the radiotherapy period in the acupuncture cohort and within the past week in the standard care cohort. ^2^Including the variables seen in table 1 and 2. SD = Standard Deviation.

**Table 2:** Personal characteristics of the patients in the verum acupuncture, sham acupuncture or standard care group.

Characteristics	Acupuncture cohort n = 215		Standard care cohort n = 62	Experiencing nausea n = 172/total n providing data = 267^1^	Univariable relative risk (95 % confidence interval)	Multivariable^2^ relative risk, (95 % confidence interval) adjusted for three groups
	Verum acupuncture n = 109	Sham acupuncture n = 106				
**Sex**, n (%)	n = 109	n = 106	n = 62	n = 267		
Man	20 (18)	15 (14)	19 (31)	35/53 (66)	1.0 (0.8–1.3)	1.0 (0.8 1.3)
Woman	89 (82)	91 (86)	43 (69)	137/214 (64)	1.0 (Ref.)	1.0 (Ref.)
Age in years: mean ± SD	64 ± 13.8	63 ±13.9	63 ± 14.5	62 ± 14.8		
19–40	7 (6)	6 (6)	6 (10)	17/19 (89)	1.5 (1.2–1.8)	1.5 (1.2–2.0)
41–60	34 (31)	34 (32)	17 (27)	55/82 (67)	1.1 (0.9–1.4)	1.1 (0.9–1.3)
61–89	68 (62)	66 (62)	39 (63)	98/164 (60)	1.0 (Ref.)	1.0 (Ref.)
**Labor status**, n (%)	n = 106	n = 104	n = 62	n = 257		
Employed	35 (33)	41 (38)	21 (34)	65/94 (69)	1.2 (1.0–1.0)	1.2 (1.0–1.4)
Retired/Sickness pension	69 (65)	59 (57)	26 (42)	82/142 (58)	1.0 (Ref.)	1.0 (Ref.)
Other	2 (2)	4 (4)	15 (24)	18/21 (86)	1.5 (1.2–1.9)	1.6 (1.1–2.1)
**Previous nausea**, n (%)						
***During previous chemotherapy***	n = 96	n = 97	n = 62	n = 256		
Not relevant	55 (57)	58 (60)	43 (69)	95/155 (61)	1.0 (0.8–1.4)	1.0 (0.8–1.4)
No	11 (11)	12 (12)	15 (24)	23/39 (59)	1.0 (Ref.)	1.0 (Ref.)
Yes	30 (31)	28 (29)	4 (6)	47/62 (76)	1.3 (1.0–1.7)	1.3 (0.9–1.7)
***During pregnancy***	n = 89	n = 92	n = 61	n = 242		
Not relevant	26 (29)	28 (30)	33 (54)	56/87 (64)	1.3 (0.9–1.7)	1.3 (1.1–1.9)
No	19 (21)	24 (26)	6 (10)	25/49 (51)	1.0 (Ref.)	1.0 (Ref.)
Yes	44 (49)	40 (43)	22 (36)	78/106 (74)	1.4 (1.1–1.9	1.4 (1.1–1.9)
***In any previous situation*** **^3^**	n = 96	n = 98	n = 61	n = 256		
No	22 (23)	29 (30)	17 (27)	30/74 (41)	1.0 (Ref.)	1.0 (Ref.)
Yes	74 (77)	69 (70)	44 (72)	134/182 (74)	1.8 (1.4–2–4)	2.0 (1.3–3.3)
***N of previous nausea situations*** **^3^**, md (25th–75th percentile)	n = 972(1–3)	n = 982(1–3)	n = 612(0–3)	n = 2572(1–3)		
0–2 situations	68 (70)	67 (68)	44 (71)	110/179 (61)	1.0 (Ref.)	1.0 (Ref.)
3–5 situations	29 (30)	31 (32)	18 (29)	56/78 (72)	1.2 (1.0–1.4)	1.2 (1.0–1.4)
**Patients'** **estimation of risk for nausea**, n (%)	n = 89	n = 94	not mea-sured	n = 183		not relevant
Lower than others	19 (21)	25 (27)		22/44 (50)	1.0 (Ref.)	
Similar to others	57 (64)	55 (59)		73/112 (65)	1.3 (1.0–1.9)	
Higher than others	13 (15)	14 (15)		22/27 (81)	1.6 (1.2–2.4)	
**Expectation of antiemetic treatment effects**, n (%)	n = 105	n = 105	not mea-sured	n = 201		not relevant
Do not believe	0 (0)	0 (0)		0/0 (0)		
Believe little	5 (5)	6 (6)		4/10 (40)	0.64 (0.3–1.4)	
Believe moderately	50 (46)	57 (54)		70/102 (68)	1.1 (0.9–1.3)	
Believe much	50 (46)	42 (40)		56/89 (62)	1.0 (Ref)	
**Previous experience of acupuncture^4^**, n (%)	n = 109	n = 101	not mea-sured	n = 209		not relevant
Yes	36 (33)	36 (34)		47/72 (65)	1.1 (0.6–1.3)	
No	73 (66)	65 (62)		82/137 (60)	1.0 (Ref)	

Numbers (n) of patients answering the questions are presented, ^1^267 of 277 patients provided data regarding nausea. Experiencing nausea was defined as any day within the radiotherapy period in the acupuncture cohort and within the past week in the standard care cohort. ^2^Including the variables seen in table 1 and 2. ^3^In travelling, unpleasant smells/sights, anxiety, chemotherapy or pregnancy. ^4^For other conditions than emesis. SD = Standard Deviation. Md = Median.

#### Nausea, vomiting and use of antiemetics

Type/dose of antiemetics and emesis during the previous 24-hours were measured by written established emesis questions [1], [17]: “Have you experienced nausea?”, answered on a four-level category scale: “No, not at all” or “Yes, a little/moderate/much” and “Have you been vomiting?” answered by “No” or “Yes”. In the acupuncture cohort the questions were asked daily during the whole radiotherapy period. In the standard care cohort, the questions were asked only once (after a mean dose of 27 Gray of radiotherapy) and at that time the questions were asked regarding the previous 24 hours and also within the time frame of the preceding week. Every patient who had experienced nausea at least once within the preceding seven days (irrespective of intensity) or vomiting was assigned to the groups “Experiencing nausea” or “Experiencing vomiting”. The emesis questions showed in pilot studies satisfactory face-validity (n = 9), construct validity (Spearman's correlation coefficient (r) 1.0; n = 456 paired observations) and test-retest reliability (r 0.98–1.0; n = 36).

#### Expectations of treatment effects and on nausea

At the end of the first, the sixth and the last verum or sham treatment the physiotherapists asked the patients: “Do you believe that the treatment that you have just received is effective in preventing or reducing nausea?” The four answer categories were “No, I do not think the treatment is effective” and “Yes, I believe a little/moderately/much that the treatment is effective”. Before treatment was started, the verum and sham treated patients answered the written question: “In relation to others, how do you estimate your own risk for becoming nauseous during the radiotherapy period?” to be answered on a five-grade category scale from “Much lower risk” to “Much higher risk”.

### Statistical analysis

The acupuncture cohort was compared with the standard care cohort using Student's t-test regarding continuous data, Mann Whitney U-test regarding ordinal or continuous, not normally distributed, data and by Fisher's exact (two categories), or Chi2-test (three categories or more), regarding category data. Relative risk (RR) for nausea with 95% confidence intervals (CI) was calculated for each of the different subgroups shown in table 1 and table 2 as compared to a reference group (RR 1.0), defined as the subgroup with the lowest prevalence of nausea. One exception was made; the subgroup of patients believing “little” in antiemetic effects of verum/sham treatment was not chosen as a reference group, because it consisted of only ten patients. A multivariable logistic regression model was constructed to determine the relative importance of the different characteristics seen in and table 2 1 for explaining the occurrence of nausea (Logistic procedure, forward selection) and the RR for nausea was adjusted in proc Genmod, with a log link and binomial error distribution. At the time that both cohorts received a mean radiotherapy dose of 27 Gray the standard care cohort was compared with the acupuncture cohort regarding occurrence of nausea and vomiting. SPSS for Windows (version 15.0.0) was used, except for calculating adjusted RR risks for nausea where we used SAS (version 9.1.3.). The significance level was set as p<0.05.

## Results

### Participants

Compared to the acupuncture cohort the standard care cohort comprised more men (p = 0.02), more patients with a testicular tumour (p = 0.001) and fewer patients consuming potent antiemetics; serotonin-receptor antagonists (p = 0.09) or corticosteroids (p<0.001) (table 1). According to the univariable analysis, nausea was not related to gender (table 2) but was more frequent in patients with testicular tumours and in patients treated with serotonin-receptor antagonists or corticosteroids (table 1). In the multivariable analysis, concomitant chemotherapy (p = 0.01, table 1), age less than 40 years (p<0.001), previous nausea in any situation (p<0.001) and a self estimated risk for nausea as higher than others during radiotherapy (p = 0.01) all indicated a significantly increased risk for nausea (table 2).

### Emesis in the verum, sham and standard care group

**Figure 2:**
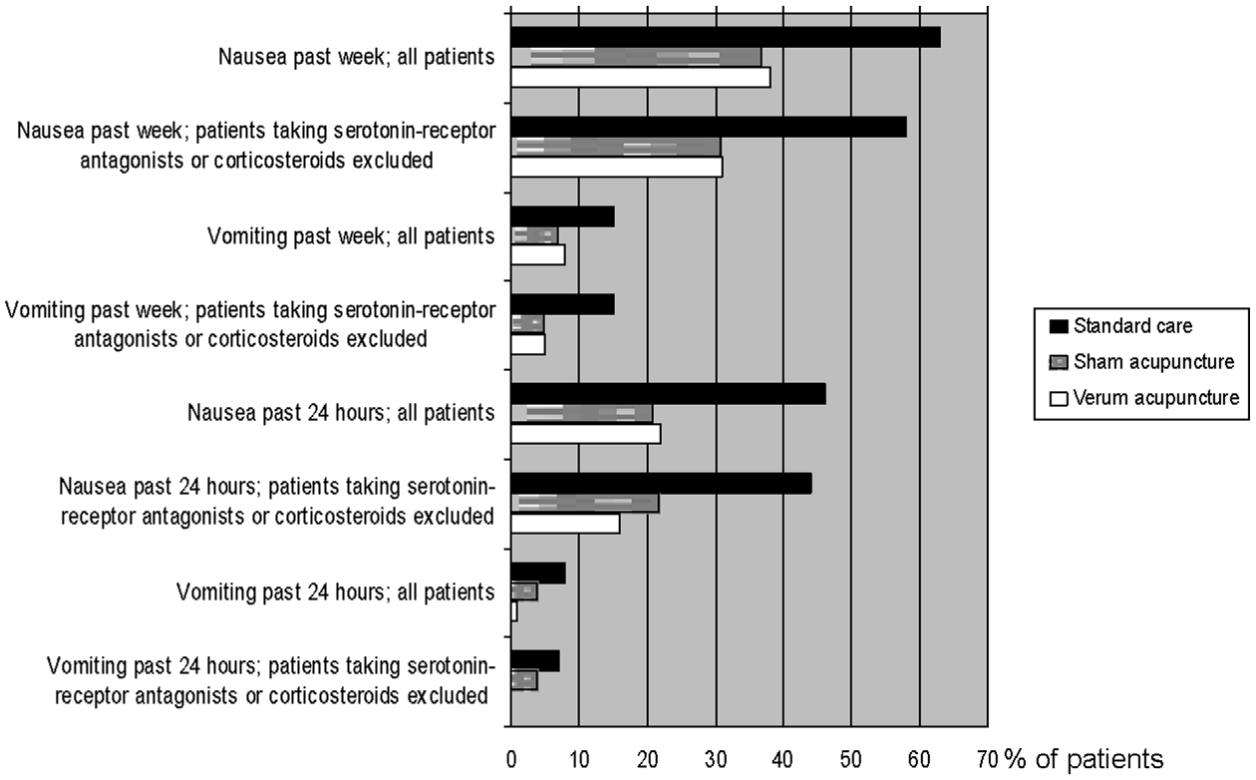
Nausea and vomiting within the past 24 hours and the past week. Emesis was measured at that time the radiotherapy dose was 27 Gray (mean) in the verum, sham and standard care groups. Measured in all patients and in patients not receiving potent antiemetics in the verum (n = 88 and n = 77), sham (n = 95 and n = 78) and standard care group (n = 62 and n = 55).

The patients in the acupuncture cohort the past week and the past 24 hours experienced significantly less occurrence of nausea and vomiting than those in the standard care cohort. The lower occurrence of nausea in the acupuncture cohort (37%) compared to the standard care cohort (63%) the past week (RR 0.6, CI 0.45–0.77) was also true when patients taking serotonin-receptor antagonists and corticosteroids were excluded (figure 2) and after adjustment for confounding factors for nausea (table 3).

**Table 3:** Comparison of nausea occurrence between the standard care cohort and the acupuncture cohort, adjusted for confounding factors for nausea.

	Nausea occurrence the past week
**Acupuncture cohort** ^1^ **,** number (%) n = 183	68 (37)
**Standard care cohort,** number (%) n = 62	39 (63)
**Relative Risk, unadjusted** (95 % Confidence Interval)	0.6 (0.5**–**0.8)
**Relative Risk, adjusted for concomitant chemotherapy** (95 % Confidence Interval)	0.3 (−0.7**–**0.7)
**Relative Risk, adjusted for age** (95 % Confidence Interval)	0.4 (−0.1**–**0.8)
**Relative Risk, adjusted for nausea in previous situations** (95 % Confidence Interval)	0.3 (−0.2**–**0.7)
**Relative Risk, overall adjustment** ^2^ (95 % Confidence Interval)	0.8 (0.6**–**0.9)

Relative risks for nausea (prevalence acupuncture cohort/ standard care cohort) during a cross sectional week of radiotherapy (mean dose 27 Gray in both cohorts). ^1^Verum and sham treated patients. ^2^Overall adjustment included adjustment for concomitant chemotherapy, age and nausea in any previous situation.

The intensity of nausea was lower in the acupuncture cohort (n 140; 78% experienced no nausea, n = 24; 13% a little nausea, n = 14; 8% moderate nausea and n = 2; 1% much nausea) than in the standard care cohort (n = 32; 52% no nausea, n = 20; 32% a little, n 9; 15% moderate and n 1; 2% much) (p = 0.002). Within the acupuncture cohort, no statistically significant differences between the verum and the sham group were seen regarding nausea occurrence or intensity, vomiting or antiemetic consumption the past 24 hours or the past week.

### Expectations of nausea and the effects of treatment in the verum and sham acupuncture groups

**Figure 3:**
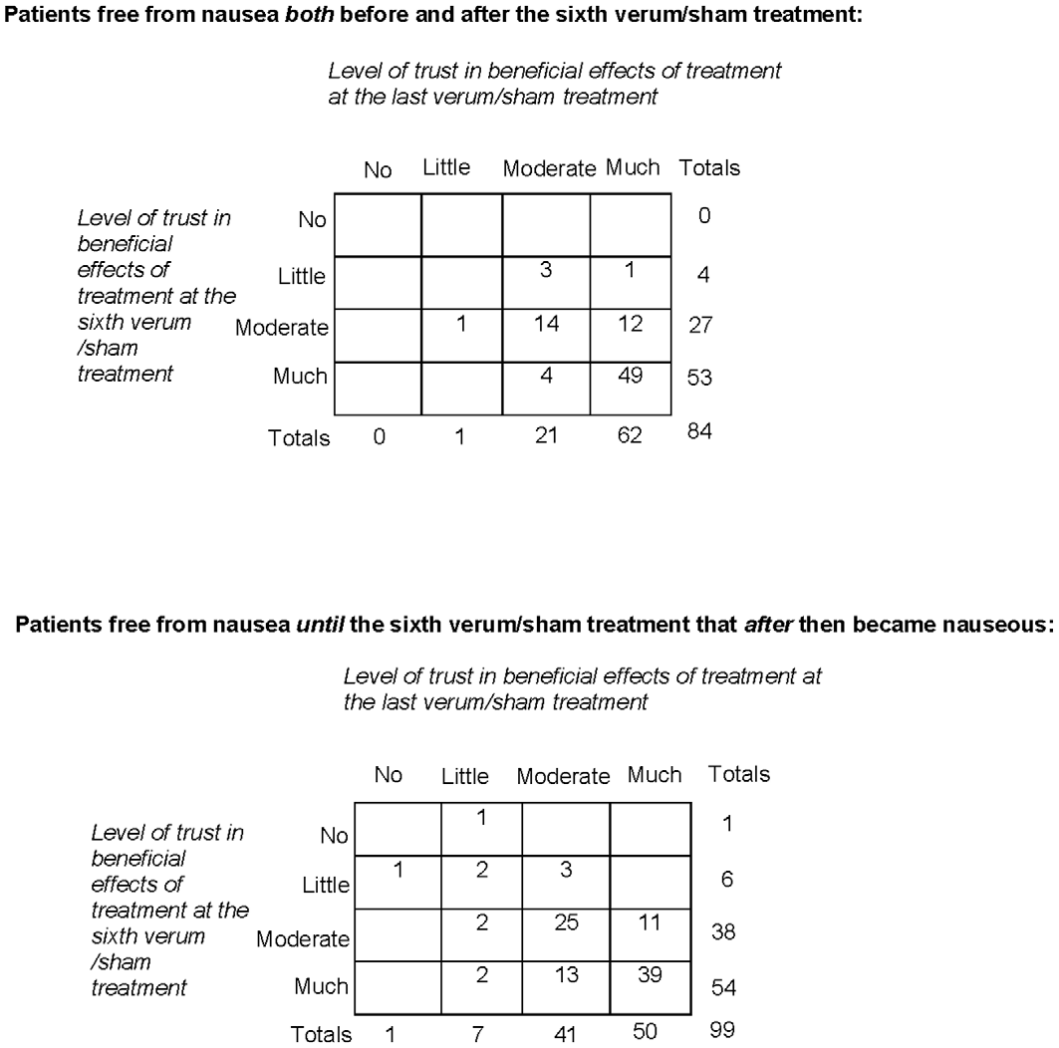
Trust in the effect of the received treatment for preventing and reducing nausea. The trust was stated at the sixth and the last verum or sham treatment in patients free from nausea and patients experiencing nausea after the sixth verum or sham treatment. Number of patients rating trust in antiemetic effects of received treatment at both the sixth and the last session was 183.

The 27 patients in the acupuncture cohort who estimated their own risk for becoming nauseous during the radiotherapy as higher than other patients had an increased risk for nausea compared to the 44 patients who estimated that they had a lower risk for nausea than others (table 2). No statistically significant differences in baseline expectations of antiemetic treatment effects were seen between the patients who experienced nausea and the patients who stayed free from nausea during the radiotherapy period (table 2). The patients who experienced nausea between the sixth and last treatments either retained or decreased their original belief in the antiemetic effects of the received treatment. The patients who stayed free from nausea either retained their original belief that the treatment would help or even reported an increase in the extent to which they trusted this treatment (figure 3). Of the patients mostly treated by therapist A (performing 1412 of 1700 treatments, 83%), 20 of 69 (29%) in the verum acupuncture group and 25 of 76 (33%) in the sham acupuncture group experienced nausea the past week. In the patients mostly treated by therapist B (performing 607 of 693 treatments, 87%), corresponding figures were 13 of 41 (32%) and 10 of 29 (35%), respectively.

## Discussion

We found lower occurrence of nausea and vomiting in patients treated with penetrating “deqi”-creating acupuncture or sham acupuncture compared to patients who had received standard care. Almost all patients in the acupuncture cohort highly expected antiemetic effects from the treatment. Patients who expected nausea had increased risk for nausea compared to patients who expected low risk for nausea.

There are many, not sham-controlled studies, reporting emesis-reducing effects of acupuncture compared to standard care in chemotherapy-induced nausea [14]. Our results indicate that nonspecific factors such as the extra care or the high expectations of positive treatment effects, not the specific characteristics of verum acupuncture, reduced emesis. Alternatively, the findings could result from flaws in our non-randomized design. Since the patients were not randomized to standard care, we investigated if an imbalance of confounding factors possibly contributing to emesis may have explained the higher prevalence of emesis in the standard care group, according to the hierarchical step model [18]. The higher risk for nausea in the standard care cohort was valid also after adjusting for possible confounding factors for emesis and after omitting patients taking serotonin-receptor antagonists and corticosteroids, indicating that our findings are valid. We have not identified any previous study of the effect of verum or sham acupuncture compared to standard care on radiotherapy-induced emesis (Pubmed, http://www.ncbi.nlm.nih.gov/ pubmed/accessed 10/11/10, using the combined search terms acupuncture, radiotherapy, nausea and vomiting). A Cochrane review of acupuncture for chemotherapy*-*induced nausea included eleven studies [14]. Only two sham-controlled studies were reported, one positive (n = 104) [19] and one negative (n = 80) [12], except for a pilot study including only 10 patients [20]. As concerns conditions in general, there exist positive sham controlled studies, but there are also indications that the effect of acupuncture may not be related to the specific characters of verum acupuncture. In line with our results, Haake and co-workers [21] found substantial improvement of back pain in 48% of 387 patients treated by verum acupuncture, in 44% of 387 patients treated with sham and in 27% of 388 patients receiving standard care. In other studies sham acupuncture reduced musculoskeletal arm pain significantly more than verum acupuncture [22].

The verum and the sham group received extra care compared to the standard care group, which may have reduced emesis: patient-therapist communication, the knowledge that continuous contact with one single therapist would continue during the whole radiotherapy period, the tactile stimulation from the therapists' hands, the extra time for rest and relaxation and the extra attention to the patient's symptoms through the daily emesis questions all are important elements of this extra care. The verum and sham performing therapists in our study might have had a supportive attitude. Arving and co-workers [23] found that chemotherapy patients who received supportive conversations reported higher quality of life and less nausea than did patients receiving standard care. Börjeson and co-workers [24] also found that chemotherapy patients who received extra care comprising information and relaxation training increased their well-being compared to patients receiving antiemetics only, despite the fact that the extra-care group received a less optimal antiemetic treatment.

Both verum acupuncture and sham-acupuncture like tactile stimulation have been seen to activate the limbic system [15]; did the low dose of sensory stimulation at the non-acupuncture point reduce emesis? Kaptchuk and co-workers [25] implied that the patient-therapist meeting was more important than the needle stimulation. Of 87 patients who received sham acupuncture from an emphatic committed therapist 62% reported adequate symptom relief of irritable bowel symptoms, compared to 44% of 88 patients receiving sham acupuncture from a non-communicating therapist and 28% of 87 patients on waiting list. The verum and sham treated patients in our study received extra time for rest and relaxation and slightly more body-contact than the standard care patients, which may have reduced distress. Psychological distress has been seen as a predictor for emesis [26] and studies indicate that relaxation [27] as well as body-contact (tactile stimulation, massage) [28] may reduce nausea in cancer patients.

Since almost all patients in the acupuncture cohort expected positive antiemetic effects of the treatment, the positive expectation may be another factor that reduced emesis. Expectations are known to influence intervention outcomes in general [29]. Indeed, Pariente and co-workers [30] found with the use of positron emission tomography (PET) that when individuals were informed that the blunt sham needle they were treated with was ineffective, no activity in the pain modulating areas in the mid-brain was seen. When a credible telescopic sham needle was used that the individuals believed was effective, a large pain-modulating activity was seen. Likewise, Linde and co-workers [31] found that acupuncture and sham treated patients with headache, chronic low back pain or osteoarthritis who had high expectations on pain-reduction reported better effects than patients with low expectations. In our study there were no differences in the occurrence of nausea between the patients who believed in the antiemetic effects of treatment and those who had a lesser belief in the antiemetic effects of treatment. Either expectations about the treatment effect were not important for nausea, or the category scale used was not sufficiently sensitive, thus resulting in a “roof effect”. The patients' trust in the antiemetic effect of verum or sham acupuncture decreased if nausea occurred and increased if nausea did not occur. This finding is in concordance with results from experiments indicating that the placebo response is a short-time effect; for example if a noxious stimulation is performed after taking a placebo pill, the study subject no longer believes in the effect of the placebo pill [29]. In our study, patients who expected nausea apparently had an increased risk for nausea, in concordance with findings regarding chemotherapy-induced nausea [32]. Thus, patients are either capable of judging their own risk for nausea or the negative expectations per se produce nausea. This finding implies that health care professionals might well consider asking the patients about their expectations about experiencing nausea and might consider the information to decide on appropriate antiemetic treatment.

Since nausea was prevalent in the standard care group and nausea may be associated with a reduced quality of life [1], [33] treatment using verum or sham acupuncture may be valuable and cost-effective since positive effects do occur. A crude calculation of the cost for providing the median number of 11 verum or sham sessions lasting 30 minutes each results in a mean cost per patient of $69 USD. Two patients (median) were treated at the same time, meaning that one patient consumed three therapist hours*. In comparison, the approximate costs of the recommended dose [6] of 8 mg of a serotonin-receptor antagonists once per day during the radiotherapy period is $98 USD**.

Emesis was measured using a well-established method [17]. The standard care cohort rated emesis only once, covering the preceding week. Some patients in the standard care cohort may, by forgetfulness, have underreported emesis, compared to the acupuncture cohort, who rated emesis daily. The acupuncture cohort was compared with a reference group, not to a third randomized arm. That design requires for a thorough investigation of potential imbalance of confounding factors between groups, as discussed above, but the design may have the benefit of avoiding the impact of the data collection per se on reported emesis. Repeated measurement of emesis per se may reduce (through the so called Hawthorne effect) or increase emesis experience [34]. Young and co-workers found that emesis questions per se increased self-reported occurrence of nausea [34]. To pay extra attention to emesis through daily data collection, without performing any extra emesis-reducing treatment in this frail patient cohort, was therefore evaluated as being unethical. We presented a cross sectional comparison at the time when the mean radiotherapy dose was the same in the acupuncture and the standard care cohorts. If we instead had observed another week of the radiotherapy period, it would not have changed the conclusions of this study; the weekly proportion of patients experiencing nausea was lower in the acupuncture cohort all radiotherapy weeks (varied 22 to 44% as described previously [13]) compared to the standard care cohort. The patients who were treated by verum or sham experienced close to 50% lower occurrence of emesis compared to the patients receiving standard care. If the extra care caused the emesis reduction, this indicates that as long as the best available antiemetic treatment is offered, patients who believe that acupuncture has beneficial effects may be satisfied with treatment with verum acupuncture or non-penetrating needles, either of which produces a moment of relaxation and attention from the therapist. A next obvious step is to further study what components in the acupuncture procedures are of importance for this dramatically positive but as yet not fully understood effect, in an effort to make possible the use of those components to further increase quality of care.

* A public hospital employing a physiotherapist for three hours spends $68 USD (408 SEK to provide the mean salary value for that service according to Swedish Association of Registered Physiotherapists 2007, www.valuta.se, date 080404). Costs for needles may be approximately $0.72 USD (24 needles consumed during 12 sessions, at six US cents according to prices at www.acuprime.com, date 101109).

** Consuming one tablet at a cost of 16.25 SEK (www.fass.se) during the mean value of 36 radiotherapy days in treatment costs $98 USD (based on cost in Sweden of 850 SEK www.valuta.se, date 101109).

## Supporting Information

Protocol S1 Trial Protocol.
(0.05 MB DOC)

Checklist S1 CONSORT Checklist.
(0.22 MB DOC)

## References

[1] Enblom A, Bergius Axelsson B, Steineck G, Hammar M, Börjeson S (2009). One third of patients with radiotherapy-induced nausea consider
their antiemetic treatment insufficient.. Support Care Cancer.

[2] Lu W (2005). Acupuncture for side effects of chemoradiation therapy in cancer
patients.. Seminars in oncology nursing.

[3] IGARR (the Italian Group for Antiemetic Research in
Radiotherapy) (1999). Radiation-induced emesis: a prospective observational multicenter
Italian trial.. Int J Radiat Oncol Biol Phys.

[4] Mystakidou K, Katsouda E, Linou A, Parpa E, Kouloulias V (2006). Prophylactic tropisetron versus rescue tropisetron in
fractionated radiotherapy to moderate or high emetogenic areas: a
prospective randomized open label study in cancer patients.. Med Oncol.

[5] Feyer PC, Maranzano E, Molassiotis A, Roila F, Clark-Snow RA Radiotherapy-induced nausea and vomiting (RINV): MASCC/ESMO
guideline for antiemetics in radiotherapy: update 2009.. http://www.ncbi.nlm.nih.gov/pubmed/20697746.

[6] Aglietti L, Roila F, Tonato M, Basurto C, Bracarda S (1990). A Pilot Study of Metoclopramide, Dexamethasone, Diphenhydramine
and Acupuncture in Women Treated with Cisplatin.. Cancer Chemotherapy and Pharmacology.

[7] Xia YS, Wang JH, Shan LJ (2000). Acupuncture Plus Ear-Point Press in Preventing Vomiting Induced
by Chemotherapy with Cisplatin.. International Journal of Clinical Acupuncture.

[8] Dibble SL, Chapman J, Mack KA, Shih A (2000). Acupressure for Nausea: Results of a Pilot Study.. Oncology Nursing Forum.

[9] Roscoe JA, Morrow GR, Hickok JT, Bushunow P, Pierce I (2003). The Efficacy of Acupressure and Acustimulation Wrist Bands for
the Relief of Chemotherapy-Induced Nausea and Vomiting: A University of
Rochester Cancer Center Community Clinical Oncology Programme Multicenter
Study.. Journal of Pain and Symptom Management.

[10] Shin YH, Kim TI, Shin MS, Juon HS (2004). Effect of Acupressure on Nausea and Vomiting during Chemotherapy
Cycle for Korean Postoperative Stomach Cancer Patient.. Cancer Nursing.

[11] Molassiotis A, Helin AM, Dabbour R, Hummerston S (2007). The effects of P6 acupressure in the prophylaxis of
chemotherapy-related nausea and vomiting in breast cancer
patients.. Complement Ther Med.

[12] Streitberger K, Friedrich-Rust M, Bardenheuer H, Unnebrink K, Windeler J (2003). Effect of acupuncture compared with placebo-acupuncture at P6 as
additional antiemetic prophylaxis in high-dose chemotherapy and autologous
peripheral blood stem cell transplantation: a randomized controlled
single-blind trial.. Clin Cancer Res.

[13] Enblom A, Johnsson A, Hammar M, Onelöv E, Steineck G (2008). Acupuncture compared to placebo acupuncture in
radiotherapy-induced nausea – a randomized controlled study. In:
Enblom A. Nausea and vomiting in patients receiving acupuncture, sham
acupuncture or standard care during radiotherapy. Linköping University
Medical Dissertations No. 1088. Paper 3 pp 1–12.. http://liu.diva-portal.org/smash/record.jsf?searchId=2&pid=diva2:207705.

[14] Ezzo JM, Richardson MA, Vickers A, Allen C, Dibble SL (2006). Acupuncture-point stimulation for chemotherapy-induced nausea or
vomiting.. Cochrane Database Syst Rev.

[15] Hui KK, Liu J, Marina O, Napadow V, Haselgrove C (2005). The integrated response of the human cerebro-cerebellar and
limbic systems to acupuncture stimulation at ST 36 as evidenced by
fMRI.. Neuroimage.

[16] Park J, White A, Lee H, Ernst E (1999). Development of a new sham needle.. Acupunct Med.

[17] Börjeson S, Hursti TJ, Peterson C, Fredrikson M, Fürst CJ (1997). Similarities and differences in assessing nausea on a verbal
category scale and a visual analogue scale.. Cancer Nurs.

[18] Steineck G, Hunt H, Adolfsson J (2006). A hierarchical step-model for causation of bias-evaluating cancer
treatment with epidemiological methods.. Acta Oncol.

[19] Shen J, Wenger N, Glaspy J, Hays RD, Albert PS (2000). Electroacupuncture for control of myeloablative
chemotherapy-induced emesis: A randomized controlled trial.. JAMA.

[20] Dundee JW, Ghaly RG, Fitzpatrick KT, Abram WP, Lynch GA (1989). Acupuncture prophylaxis of cancer chemotherapy-induced
sickness.. J R Soc Med.

[21] Haake M, Müller HH, Schade-Brittinger C, Basler HD, Schäfer H (2007). German Acupuncture Trials (GERAC) for chronic low back pain:
randomized, multicenter, blinded, parallel-group trial with 3
groups.. Arch Intern Med.

[22] Goldman RH, Stason WB, Park SK, Kim R, Schnyer RN (2008). Acupuncture for treatment of persistent arm pain due to
repetitive use: a randomized controlled clinical trial.. Clin J Pain.

[23] Arving C, Sjödén PO, Bergh J, Hellbom M, Johansson B (2007). Individual psychosocial support for breast cancer patients: a
randomized study of nurse versus psychologist interventions and standard
care.. Cancer Nurs..

[24] Börjeson S, Hursti TJ, Tishelman C, Peterson C, Steineck G (2002). Treatment of nausea and emesis during cancer chemotherapy.
Discrepancies between antiemetic effect and well-being.. J Pain Symptom Manage.

[25] Kaptchuk TJ, Kelley JM, Conboy LA, Davis RB, Kerr CE (2008). Components of placebo effect: randomised controlled trial in
patients with irritable bowel syndrome.. BMJ.

[26] Zachariae R, Paulsen K, Mehlsen M, Jensen AB, Johansson A (2007). Chemotherapy-induced nausea, vomiting, and fatigue–the role
of individual differences related to sensory perception and autonomic
reactivity.. Psychother Psychosom.

[27] Luebbert K, Dahme B, Hasenbring M (2001). The effectiveness of relaxation training in reducing
treatment-related symptoms and improving emotional adjustment in acute
non-surgical cancer treatment: a meta-analytical review.. Psychooncology.

[28] Myers CD, Walton T, Bratsman L, Wilson J, Small B (2008). Massage modalities and symptoms reported by cancer patients:
narrative review.. J Soc Integr Oncol.

[29] Price DD, Finniss DG, Benedetti F (2008). A comprehensive review of the placebo effect: recent advances and
current thought.. Annu Rev Psychol.

[30] Pariente J, White P, Frackowiak RS, Lewith G (2005). Expectancy and belief modulate the neuronal substrates of pain
treated by acupuncture.. Neuroimage.

[31] Linde K, Witt CM, Streng A, Weidenhammer W, Wagenpfeil S (2007). The impact of patient expectations on outcomes in four randomized
controlled trials of acupuncture in patients with chronic
pain.. Pain.

[32] Higgins SC, Montgomery GH, Bovbjerg DH (2007). Distress before chemotherapy predicts delayed but not acute
nausea.. Support Care Cancer.

[33] Shun SC, Chiou JF, Lai YH, Yu PJ, Wei LL (2008). Changes in quality of life and its related factors in liver
cancer patients receiving stereotactic radiation therapy.. Support Care Cancer.

[34] Young SD, Adelstein BD, Ellis SR (2007). Demand characteristics in assessing motion sickness in a virtual
environment: or does taking a motion sickness questionnaire make you
sick?. IEEE Trans Vis Comput Graph.

