# Supplementary material for: Getting the Grip on Nonspecific Treatment Effects: Emesis in Patients Randomized to Acupuncture or Sham Compared to Patients Receiving Standard Care
Source: PLoS One. 2011 Mar 23;6(3):e14766. doi: 10.1371/journal.pone.0014766 (PMC3063156; doi:10.1371/journal.pone.0014766)
Supplement: Protocol S1 — Trial Protocol. (0.05 MB DOC) [file pone.0014766.s001.doc]

**Acupuncture Versus Sham for Radiotherapy-Induced Emesis**

**This study has been completed.**

Study NCT00621660.   Last updated on February 21, 2008. http: www.clinicaltrial.gov

Information provided by University Hospital, Linkoeping

**This Tabular View shows the required WHO registration data elements as marked by ^†^**

| **Descriptive Information Fields** | |
| --- | --- |
| **Brief Title ^†^** | Acupuncture Versus Sham for Radiotherapy-Induced Emesis |
| **Official Title ^†^** | Acupuncture Compared To Sham With a Placebo-Needle in Radiotherapy-Induced Nausea - a Randomised Controlled Study |
| **Brief Summary** | The aim of this study is to evaluate if acupuncture prevents or reduces nausea or vomiting during radiotherapy |
| **Detailed Description** | Treatment with acupuncture is, despite sometimes unclear evidence, increasing in cancer care. Acupuncture is used for indications such as pain and nausea, but for radiotherapy (RT) induced nausea it is still an unexplored treatment. For evaluation of the method, the use of sham acupuncture as a control treatment provides a tool resembling placebo for drugs. The aim of the studt is therefore to investigate whether acupuncture reduces nausea caused by radiotherapy in a patient group with a >50% risk of experiencing the symptoms (abdominal or pelvic region). Patients are randomised to invasive acupuncture (IA) or placebo acupuncture (PA) 30 min, 2-3 times/week during the whole RT period. IA is administered bilaterally to the point PC6 using an invasive needle and PA with a needle, which looks identical but is not pointed and is not fixed in its handle. When this comes into contact with the surface of the skin and gives a feeling of penetration it glides upwards in its handle and is therefore shortened, which gives an illusion that the needle has entered the tissue. Nausea and vomiting is documented in diaries and questionnaires under the entire treatment period as well as two and four weeks after radiotherapy. |
| **Study Phase** |  |
| **Study Type ^†^** | Interventional |
| **Study Design ^†^** | Treatment, Randomized, Double Blind (Subject, Investigator, Outcomes Assessor), Placebo Control, Parallel Assignment, Efficacy Study |
| **Primary Outcome Measure ^†^** | Number of patients with at least one episode of nausea during the whole radiotherapy treatment period [ Time Frame: The radiotherapy treatment period (md 5 weeks) ] [ Designated as safety issue: No ] |
| **Secondary Outcome Measure ^†^** | Secondary outcome measures are the summed number of days with nausea, intensity of nausea, number of patients and summed number of days with vomiting, belief in the antiemetic effects and interest in receiving needling in the future [ Time Frame: From acupunture start until 4 weeks after treatment stopped ] [ Designated as safety issue: Yes ] |
| **Condition ^†^** | Nausea Vomiting |
| **Intervention ^†^** | Procedure: Acupuncture Procedure: Sham |
| **MEDLINE PMIDs** |  |
| **Links** |  |
|  |  |
| **Recruitment Information Fields** | |
| **Recruitment Status ^†^** | Completed |
| **Enrollment ^†^** | 237 |
| **Start Date ^†^** | January 2004 |
| **Completion Date** | March 2007 |
| **Primary Completion Date** | March 2007   (final data collection date for primary outcome measure) |
| **Eligibility Criteria ^†^** | Inclusion Criteria:   - patients of at least 18 years of age - with gynaecologic-, anal-, rectal-, colon-, ventricle-, pancreatic- or testicular tumours - willing to give their informed consent - able to take part in the entire treatment and data collection procedure - had planned radiation over an abdominal and/or pelvic field (with or without concomitant chemotherapy) with the volume of at least 800 cm3 and a dose of at least 25 Gy.   Exclusion Criteria:   - use of antiemetic treatment or persistent nausea within 24 hours prior to the start of radiotherapy - ever received acupuncture against nausea, or during the last year received acupuncture for any indication. |
| **Gender** | Both |
| **Ages** | 18 Years and older |
| **Accepts Healthy Volunteers** | No |
| **Contacts ^††^** |  |
| **Location Countries ^†^** | Sweden |
|  |  |
| **Administrative Information Fields** | |
| **NCT ID ^†^** | NCT00621660 |
| **Organization ID** | 4960-B04-01XAC |
| **Secondary IDs ^††^** | 02-420, M167-04 |
| **Study Sponsor ^†^** | University Hospital, Linkoeping |
| **Collaborators ^††^** | The Swedish Cancer Society The County Council of Östergötland The Vardal Institute Cancer & Traffic Injury Fund |
| **Investigators ^†^** | | Principal Investigator: | Sussanne Börjeson, PhD | Linköping University |  | | --- | --- | --- | --- | |
| **Information Provided By** | University Hospital, Linkoeping |
| **Verification Date** | November 2007 |
| **First Received Date ^†^** | February 11, 2008 |
| **Last Updated Date** | February 21, 2008 |

^†^   Required WHO trial registration data element.
^††^   WHO trial registration data element that is required only if it exists.
